# Editorial: Radical cystectomy in bladder cancer patients

**DOI:** 10.3389/fonc.2022.1113112

**Published:** 2022-12-19

**Authors:** Vadim S. Koshkin, Nima Almassi, Jeanny B. Aragon-Ching

**Affiliations:** ^1^ Helen Diller Family Cancer Center, University of California San Francisco, San Francisco, CA, United States; ^2^ Center for Urologic Oncology, Department of Urology, Cleveland Clinic, Cleveland, OH, United States; ^3^ Inova Genitourinary (GU) Cancer Program, Inova Schar Cancer Institute, Fairfax, VA, United States; ^4^ Department of Medicine, University of Virginia, Charlottesville, VA, United States

**Keywords:** bladder cancer, radical cystectomy, biomarkers, urothelial cancer (UC), nomogram, overall survival

Bladder cancer accounts for 3% of global cancer diagnoses (1) and is especially prevalent in the developed world, being responsible for close to 212,000 deaths worldwide annually. Radical cystectomy, preceded by neoadjuvant chemotherapy for eligible patients, remains the standard of care and the curative option for patients with muscle-invasive bladder cancer. However, biomarkers predictive of outcomes with radical cystectomy have remained elusive. In this issue of *Frontiers in Oncology* focusing on radical cystectomy in bladder cancer patients, several manuscripts describe the prognostic value of different biomarkers, including the use of a systemic inflammatory response index in patients undergoing radical cystectomy by Ni et al., a combination of a c-reactive protein (CRP) and neutrophil-to lymphocyte ratio (NLR) as a novel prognostic index by Wang et. al., evaluating an immunohistochemical signature by Wu et. al., and the preoperative serum gamma-glutamyltransferase by Su et al.

In the paper by Ni et al., authors graded a systemic inflammatory response index (SIRI) in patients with bladder cancer (BCa) and determined that severity of grade correlated with disease-free survival (DFS) and overall survival (OS). Furthermore, the proposed nomogram performed better in predicting outcomes compared to the usual AJCC TNM staging. While systemic inflammatory response and inflammation have historically been tied to cancer growth, proliferation and metastasis, and theories abound as to the role of indirect inflammatory biomarkers or hematologic-based indices, nomograms that will be used in clinical practice post-cystectomy also require validation to be clinically meaningful and applicable in routine practice. Other efforts to further define prognostic markers come in the form of c-reactive protein and the NLC ratio which have also been a topic of interest by numerous investigators. Wang et al. investigated the role of preoperative C-NLR (C-reactive protein and neutrophil-to-lymphocyte ratio; NLR), which is a novel systemic inflammation index combining the two inflammatory markers. They studied the relationship of this novel systemic inflammation index to AJCC TNM staging as predictor of outcomes including OS and DFS and also how it performs in the nomogram. The authors found good concordance and accuracy in using the classification of low C-NLR, consisting of the majority of their 199 eligible patients with BCa (58%) as opposed to 42% of patients classified high C-NLR. While the nomogram stays accurate with good receiving operating characteristics (ROC) curves, validation in a bigger population would be important for clinical application.

In the paper by Wu et al., they conducted a LASSO Cox regression model analysis and developed an immunohistochemistry (IHC)-based classifier that looked at 9 prognostic markers (HER2, EGFR, VEGF, CyclinD1, BAX, MDR, p53, p27, and TOPOII) in the training cohort. There were 366 bladder cancer patients of whom the majority (71%) had muscle-invasive bladder cancer (MIBC) and 24% had lymph node metastasis, but none had distant metastatic disease. The findings suggest that correlation between survival outcomes of DFS was high with the severity of risk, such that the 10-year DFS in the low-risk patients was better (71%) compared with high-risk patients (18%) (p < 0.001). The authors did have a validation cohort which showed similar findings of equivalent 10-year DFS of 86% for the low-risk group and 20% for the high-risk group (p < 0.001). Authors concluded that this IHC-based classifier of nine markers is a reliable prognostic tool which can eventually guide clinical decision making regarding treatment strategy. At the current time, the role of targeted therapy in bladder cancer is limited to the metastatic space (2). Therefore, while this dataset can inform future studies, clinical application is unlikely to be ready for standard use prior to the emergence of prospective data.

The final manuscript on biomarkers was a study by Su et al., which examined the role of serum gamma-glutamyltransferase (GGT) which is considered a membrane bound enzyme, with a key role in glutathione metabolism that is important for cellular protection against oxidants. With oxidative stress in the tumor microenvironment, GGT has been found to be elevated and perhaps play a role in various cancer resistance, development and progression (3). This paper examined 263 patients with bladder cancer and correlated serum GGT with phenotypic characteristics and survival. They found good correlation and prediction of elevated GGT with rate of OS, DFS and cancer-specific survival (CSS) (p<0.001 for all). These results are preliminary, and given the non-specific nature of this biomarker, also unlikely to be immediately clinically applicable.

Surgical treatment of bladder cancer also requires extensive surgical expertise. The paper by Chen et al., explored the prognostic value of the log odds of negative lymph nodes/T stage (LONT) and constructed a nomogram based on data from the Surveillance, Epidemiology, and End Results (SEER) from 2004 to 2015. They utilized internal patients to serve as the validation cohort. Their findings suggest that LONT was an independent and significant prognostic factor with good ROC and prediction of CSS at 3 and 5 years with 0.783 and 0.774 in the primary cohort and 0.781 and 0.781 in the validation cohort. These findings are consistent with prior studies demonstrating an association between lymph node yield and oncologic outcomes, and highlight the prognostic and potentially therapeutic benefit of a thorough lymph node dissection at radical cystectomy, something that is currently being investigated in prospective randomized trials. This data perhaps carries even more relevance in the era of adjuvant nivolumab therapy which has shown benefit in patients with high risk of recurrence, such as patients with node-positive disease. The final manuscript in this edition of Frontiers is the publication by Noh et al. This observational study demonstrates a single-surgeon learning curve with implementation of robotic radical cystectomy with intracorporeal urinary diversion, demonstrating decreasing operative time and higher likelihood of achieving the “pentafecta” of optimal oncologic and perioperative outcomes with increasing surgical experience. Unlike other robotic cystectomy series, the majority of patients in this series received continent urinary diversions despite the greater complexity of this diversion with the intracorporeal approach. As robotic cystectomy is increasingly adopted, it is important that selection of the type of urinary diversion is determined by patient preferences and candidacy rather than the surgical approach employed.

In summary, this collection highlights the role of emerging biomarkers and application of nomograms in predicting clinical outcomes including OS, DFS and CCS in patients with bladder cancer undergoing radical cystectomy. This continues to be a rapidly evolving field and while there have been many successes, bladder cancer remains a very challenging disease to treat. A significant proportion of patients with muscle-invasive urothelial cancer will unfortunately succumb to their disease, which makes further diagnostic, prognostic and treatment advances–and further studies identifying biomarkers of response–a priority.

## Author contributions

VK, NA, JA-C drafted, reviewed, edited, finalized the manuscript. All authors contributed to the article and approved the submitted version.
